# Techno-Economic Analysis of 2,3-Butanediol Production from Sugarcane Bagasse

**DOI:** 10.1021/acssuschemeng.3c01221

**Published:** 2023-05-22

**Authors:** Siddharth Gadkari, Vivek Narisetty, Sunil K. Maity, Haresh Manyar, Kaustubha Mohanty, Rajesh Banu Jeyakumar, Kamal Kishore Pant, Vinod Kumar

**Affiliations:** † Department of Chemical and Process Engineering, University of Surrey, Guildford GU2 7XH, U.K.; ‡ School of Water, Energy and Environment, Cranfield University, Guildford MK43 0AL, U.K.; § Department of Chemical Engineering, Indian Institute of Technology Hyderabad, Kandi, Sangareddy, Telangana 502284, India; ∥ School of Chemistry and Chemical Engineering, Queen’s University Belfast, Belfast, Northern Ireland BT9 5AG, U.K.; ⊥ Department of Chemical Engineering, Indian Institute of Technology Guwahati, Guwahati, Assam 781039, India; # Department of Life Sciences, Central University of Tamil Nadu, Neelakudi, Thiruvarur, Tamil Nadu 610005, India; ¶ Department of Chemical Engineering, Indian Institute of Technology Delhi, New Delhi 110016, India; ▲ Department of Biosciences and Bioengineering, Indian Institute of Technology Roorkee, Roorkee, Uttarakhand 247667, India

**Keywords:** sugarcane bagasse, 2,3-butanediol, techno-economic analysis, net present value, sensitivity analysis

## Abstract

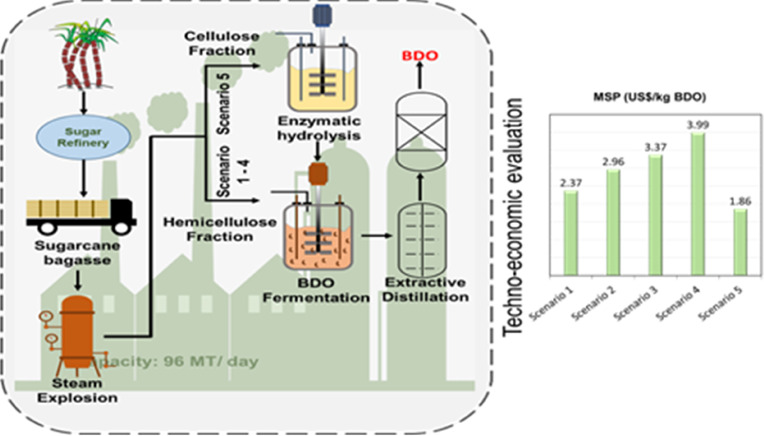

Sugarcane bagasse (SCB) is a significant agricultural residue generated by sugar mills based on sugarcane crop. Valorizing carbohydrate-rich SCB provides an opportunity to improve the profitability of sugar mills with simultaneous production of value-added chemicals, such as 2,3-butanediol (BDO). BDO is a prospective platform chemical with multitude of applications and huge derivative potential. This work presents the techno-economic and profitability analysis for fermentative production of BDO utilizing 96 MT of SCB per day. The study considers plant operation in five scenarios representing the biorefinery annexed to a sugar mill, centralized and decentralized units, and conversion of only xylose or total carbohydrates of SCB. Based on the analysis, the net unit production cost of BDO in the different scenarios ranged from 1.13 to 2.28 US$/kg, while the minimum selling price varied from 1.86 to 3.99 US$/kg. Use of the hemicellulose fraction alone was shown to result in an economically viable plant; however, this was dependent on the condition that the plant would be annexed to a sugar mill which could supply utilities and the feedstock free of cost. A standalone facility where the feedstock and utilities were procured was predicted to be economically feasible with a net present value of about 72 million US$, when both hemicellulose and cellulose fractions of SCB were utilized for BDO production. Sensitivity analysis was also conducted to highlight some key parameters affecting plant economics.

## Abstract 2

Sustainable production of 2,3-butanediol using different carbon fractions of sugarcane bagasse and its economic feasibility are studied.

## Introduction

The gradual depletion of fossil resources and the associated environmental pollution problems are posing a severe threat to the world. Industrial biotechnology making use of microbial cell factories has emerged as a potential alternative to the petrochemical route for sustainable manufacturing of building block chemicals. Though the production of the platform chemicals from first-generation biomass has been very successful, it gives rise to the food versus feed debate. The biological route provides the opportunity for using both non-edible as well as waste biomass, which leads to reduced waste generation with simultaneous production of platform chemicals and achieving the goal of a carbon-neutral society.^1,2^ Lignocellulosic biomass is the most abundant waste biomass on earth and contains cellulose, hemicellulose, and lignin as major fractions. Cellulose and hemicellulose are inexpensive sources of fermentable sugars. Cellulose is a homopolymer of glucose, while hemicellulose is a heteropolymer and contains a mixture of C5 (xylose and arabinose) and C6 (glucose, galactose, and mannose) sugars. Xylose is the major monosaccharide in hemicellulose, making it the second most abundant sugar in lignocellulosic biomass after glucose. The depolymerization of these two polysaccharides generates sugar platform for sustainable biorefineries.^1,3^

Sugarcane is one of the most cultivated crops across the world to meet sugar and liquor demands. It is grown in more than 100 countries with an annual production of 1907 million MTs. Brazil and India are the leading producers of sugarcane in the world. The two main products coming from sugarcane mills are sugar (sucrose) and ethanol, while the major waste stream is sugarcane bagasse (SCB) which is a dry fibrous residue obtained after the extraction of juice from sugarcane. Crushing one MT of sugarcane generates approximately 0.3 metric MTs of SCB, leading to an annual global production potential of ∼570 million MTs. SCB is thus one of the largest agricultural residues in the world. The current practices by sugar mills, particularly in India, involve burning SCB to generate heat and electricity for the plant. SCB is lignocellulosic biomass with the following composition, cellulose: 40–50%; hemicellulose: 25–35%; and lignin: 20–30%.^4−8^ Being a rich source of fermentable sugars, SCB can be valorized to high-value products with a circular biorefining approach. Like other lignocellulosic feedstocks, the majority of the literature has focused on using cellulosic sugars from SCB for fermentative production of fuels and chemicals, with limited research on the hemicellulose fraction rich in xylose.^1^

One such high-value chemical is 2,3-butanediol (BDO), which is a straight chain C4 diol with hydroxyl groups attached to the second and third carbon atom. BDO finds a multitude of applications in food, pharmaceutical, and chemical industries and shows potential as a biofuel due to its high heat of combustion. BDO is a gateway molecule to a variety of chemical products with vast commercial potential. The total market of BDO and its derivatives is estimated to be ∼$43 billion. Both BDO and *n*-butanol are C4 alcohols. However, low solvent titer (∼20 g/L) together with complex acetone–butanol–ethanol (ABE) separation is the major challenge involved in ABE fermentation, making bio-*n*-butanol highly expensive than the petrochemical route. On the contrary, the fermentative route has been reported to achieve a much high BDO titer (>100 g/L), making it an ideal candidate for manufacturing from renewable sources.^9,10^ Despite this, even today, the dominant route for BDO production is the petrochemical one. The current market price of BDO is ∼$2.8–3.5/kg, and a cheap production via the microbial route can be cost competitive to fossil-based production.^11−13^ One of the possible solutions to circumvent the high production cost is to manufacture BDO from low-cost feedstock, such as SCB, brewer’s spent grain (BSG), sugar beet pulp, bread waste, etc. For example, Amraoui and associates achieved an accumulation of 118.5 g/L BDO using the cellulosic fraction of BSG with yield and productivity of 0.43 g/g and 1.65 g/L h, respectively.^10^ In another report, Amraoui and associates used detoxified xylose-rich hydrolysate from SCB for BDO production and reported a BDO titer of 63.5 g/L with yield of 0.36 g/g.^14^ The results were comparable to pure xylose, where 71.1 g/L BDO was achieved with a conversion yield of 0.40 g/g. Furthermore, the BDO obtained from pure xylose and detoxified SCB hydrolysate was extracted and recovered by the aqueous two-phase system method using isopropanol as the extractant and (NH_4_)_2_SO_4_ as a salting-out agent. The recovery of BDO in both cases was more than 85%. In a recent work, Narisetty and associates generated BDO from bread waste and amassed 138.8 g/L titer with yield and productivity of 0.48 g/g and 1.45 g/L h, respectively.^2^ All these reports indicate that using non-edible and waste biomass can result in developing an industrial process for BDO manufacturing.

The prospect of microbial production of BDO industrially, as suggested by these experimental studies, necessitates validation by detailed techno-economic performance analysis of the proposed processes. Techno-economic analysis (TEA) is an effective and standard methodology to evaluate the economic feasibility of a product or process. TEA has been previously reported for utilizing SCB as a feedstock in the production of liquid biofuels, lactic acid, succinic acid, bioethanol, furfural, biosurfactant, xylitol, activated carbons, or simply for energy generation.^15−25^ Munagala et al. (2021) described the overall sustainability of SCB valorization to produce lactic acid based on the life cycle and TEA of biorefinery annexed to a sugar mill.^23^ Shaji et al. (2021) also focused on utilizing SCB and performed a detailed sustainability assessment of a biorefinery producing succinic acid from SCB.^21^ Studies by Mesa et al. (2016), Gubicza et al. (2016), and Ntimbani et al. (2021) have described TEA of ethanol production using SCB.^18,19,22^ There are some studies describing the economic feasibility of BDO production from various carbon sources, such as glycerol, sucrose, sugarcane molasses, BSG, food waste, and lignocellulosic biomass.^12,13,26,27^ TEA by Koutinas et al. (2016) focused on the estimation of the minimum selling price (MSP) of BDO using glycerol, sucrose, and sugarcane molasses. This study reported that the complex nutrient supplements, raw material market price, and fermentation efficiency were major contributors to MSP.^13^ Haider et al. (2018) conducted a techno-economic evaluation of BDO production from fermentation broth based on four different distillation designs for separation and purification.^28,29^ Harvianto et al. (2018) also compared the economic feasibility of BDO separation from fermentation broth based on conventional distillation with that of a hybrid extraction-distillation (HED) scheme. It was shown that adding an extraction column before distillation improved the process economics.^30^ Mailaram et al. (2022) described TEA of BDO production using C5 and C6 sugars derived from BSG.^26^ They used the pinch technology as a process integration tool to minimize the external utility consumption and calculated the unit production cost and MSP for different BDO titers and plant capacities corresponding to the centralized and decentralized biorefinery. However, to the best of our knowledge, there is no study on TEA to produce BDO using SCB as feedstock.

The current study has been carried out to evaluate the economic feasibility of fermentative BDO production from SCB. The technical feasibility of this process has been established experimentally, as described in our recent papers,^14,31^ but before further investment in pilot-scale studies could be made, it will be critical to investigate if this process can lead to cost-effective production of BDO and profitability of the proposed biorefinery system.

## Methodology

In this study, a 96 MT/day SCB processing plant was modeled to evaluate the techno-economic performance of BDO production using SuperPro Designer process simulator (version 12, Build 03). Experimental data from our previous studies were used to describe mass and energy balances in the different stages of product formation, such as pre-treatment, fermentation, and subsequent downstream processing (DSP). Design specifications with appropriate thermodynamic property methods were used to model auxiliary processes, such as grinding, washing, screw-pressing, steam, and power generation. This work proposes to build the plant in India as an annexure to an existing sugar mill that produces SCB waste. The construction phase of the plant will be one year with a start-up period of three months. The operating lifetime of the plant was assumed to be 20 years, with 312 operating days every year and the remaining days for maintenance. Specific details of the production process are explained below.

### Process Description

**Figure 1:**
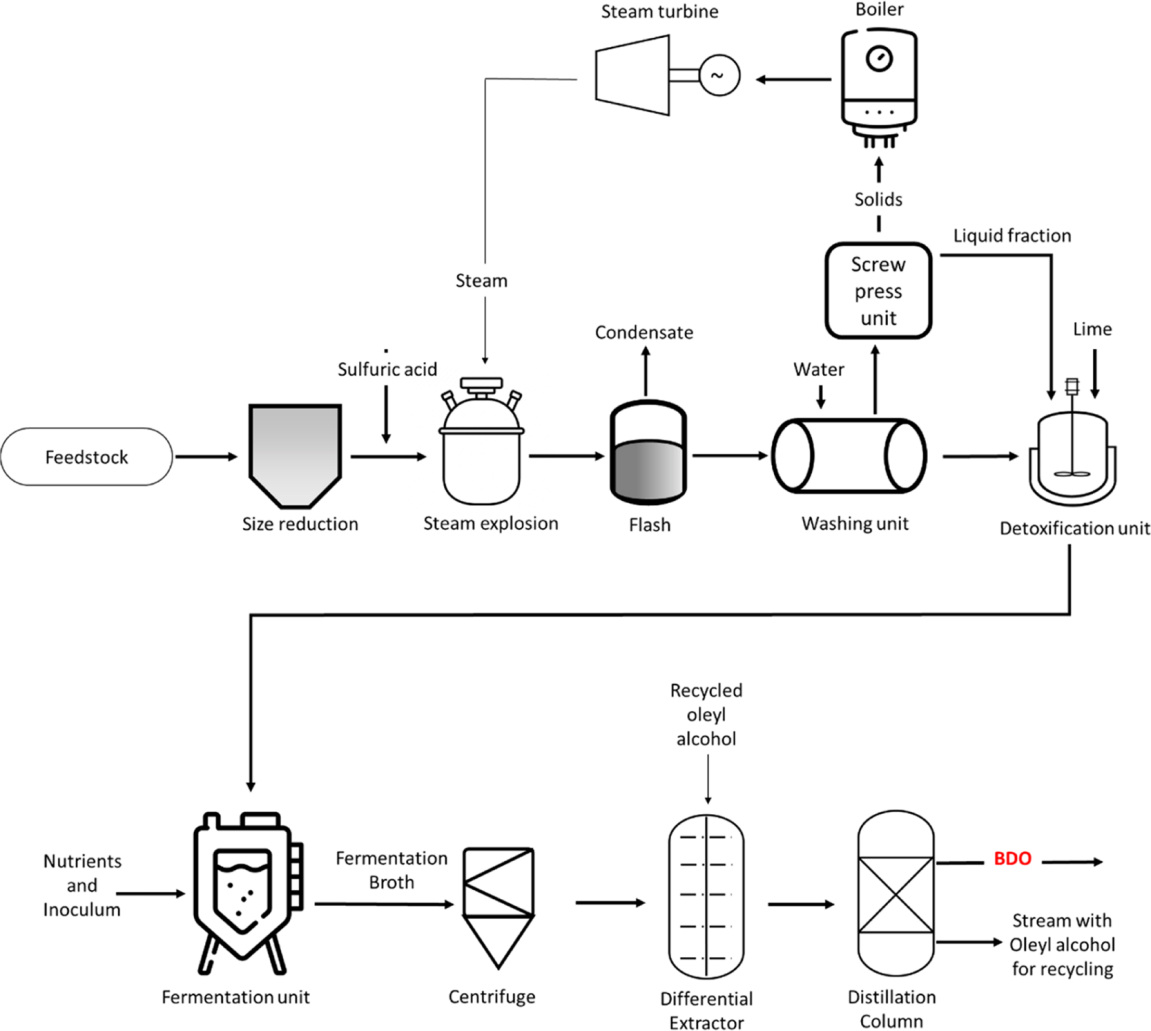
Simplified scheme of the BDO production plant modeled in this study.

The BDO production process from SCB-derived xylose was reported in our previous publications.^14,31^ A simplified scheme of the proposed plant is depicted in Figure 1. Analysis assumes that the moisture content in the feedstock was 35%, and the chemical composition of dry SCB was as follows: cellulose 48.5%, hemicellulose 21%, lignin 18%, ash 3.4%, and other solids 9.1%.^25^ The process starts with size reduction of SCB, followed by dilute acid pre-treatment using sulfuric acid (5% w/w) and subsequent steam explosion. The pre-treatment step converts hemicellulose to xylose, leaving the cellulose and lignin fractions mostly intact. The conversion percentages vary among the different studies.^32,33^ In this analysis, we have assumed 100% conversion of hemicellulose to xylose with trace amounts of other sugars and acetic acid. We also considered 10% conversion of cellulose to glucose and 1% conversion of xylose obtained from hemicellulose to furfural. In all these reactions, we have kept the final xylose concentration and mass flow rate consistent with the pre-treatment data obtained from Nova Pangaea Technology Limited, who have developed and optimized the pre-treatment process for treating 4 dry MT/h of SCB.^25^ The output from pre-treatment at high pressure is sent to a flash vessel and then to a washer unit where wastewater containing minor impurities and organic acids are separated. The other stream is sent to a screw press unit, which separates xylose in the liquid form from the solid residue containing remaining cellulose and lignin. The solid cake is then forwarded to a boiler for steam generation, while the xylose-rich stream is sent to a detoxification unit to remove any remaining impurities. Detoxified xylose-rich hydrolysate derived from SCB is then transferred to a fermenter for BDO production. An adequate quantity of mutant strain of *Enterobacter ludwigii* (10% v/v) was first sent to a seed fermentation unit with the supply of essential nutrients before transferring to the fermenter.

The BDO concentration was 63.5 g/L in the broth output from the fermenter, and it was fed to a centrifugation unit for solid–liquid separation. The reaction stoichiometry for xylose and glucose conversion to BDO is as follows:

Xylose to BDO
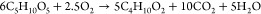
1

Glucose to BDO
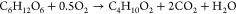
2

The carbon loss in the form of CO_2_ during BDO fermentation is inevitable, and besides, a substantial amount of carbon is lost in the form of byproducts. In our previous work, ∼30% carbon was lost in the form of CO_2_ largely generated during biosynthesis of BDO and acetoin, and about 22–24% was lost due to the byproduct formation including ethanol, acetic, succinic, and lactic acid.^14,34^ For TEA, we have not considered the formation of byproducts in our analysis; however, the BDO yield is considered in the process model, which forms the basis of our work and does take care of the carbon loss in form of CO_2_ and byproducts.

The HED separation method, as described by Harvianto et al. (2018), was used for downstream separation and purification of BDO from the fermentation broth. Liquid stream from the centrifuge was first sent to an extractor unit, where it was mixed with a solvent.^30^ Based on the study by Harvianto et al. (2018), oleyl alcohol, which has high selectivity for BDO, was used as the solvent in this work. BDO was extracted to the solvent-rich organic phase, which was separated from the water-rich phase by decantation.^30^ The organic phase leaving the extractor was sent to a distillation column, where BDO was recovered as the distillate and the solvent as the bottom product. The solvent was recycled back to the extraction column (10% loss is assumed). Here, it should be noted that the impact of large amount of unreacted sugars in the performance of the hybrid extraction distillation system is not entirely known, and how this would affect the efficiency of the extraction and the purity of the product would need to be evaluated based on a comprehensive experimental study. The extractant used in our study, i.e., oleyl alcohol, demonstrated a high BDO distribution coefficient and low water solubility. The high distribution coefficient ensured the maximum recovery of BDO from fermentation broth during extraction. Our extraction process model showed that more than 99% BDO could be recovered. The oleyl alcohol has low water solubility and vice versa. Therefore, the organic and aqueous phases contained only a small amount of water and oleyl alcohol, respectively. On the other hand, the unreacted sugars, being highly polar with high water solubility, remained mostly in the aqueous phase. Therefore, we selected the oleyl alcohol as an extractant for the extraction of BDO from the fermentation broth, from which pure BDO was recovered by distillation.

### Scenarios

In this work, fermentative BDO production from SCB-derived xylose was studied considering five different process scenarios. These scenarios will eventually help us determine the cut-off for the plant becoming profitable and the range of economic feasibility.

1. The first scenario (called the base case) assumes that SCB is obtained free of cost. It also assumes that all the utilities required in the process are generated in the same combined facility. Part of the steam requirement is fulfilled from the co-generating steam by burning the solid residue obtained from the screw-press unit during pre-treatment, with additional requirements met by the combined heat and power units in the attached sugar mill. Cooling water requirement is also met through the facilities in the sugar mill. However, it is assumed that natural gas needs to be purchased separately.
2. Unlike the base case, the second scenario assumes that SCB is procured at the cost of US$ 50/MT.^17,23^ Other factors remain same as scenario 1.
3. The third scenario considers that the BDO production plant is no longer annexed to a sugar mill but is a standalone facility. Therefore, all costs associated with utilities that cannot be generated in-house would need to be included. In this scenario, part of the required steam is generated using the solid residue from the screw-press unit during pre-treatment in a boiler/steam generator, and the remaining is met by using additional SCB. Here, SCB is still assumed to be free.
4. The fourth scenario is a combination of the second and third scenarios, where the costs of both SCB and utilities are included. This scenario refers to a standalone facility for BDO production using SCB, which is purchased at US$ 50/MT.
5. The fifth scenario builds up on the fourth scenario. This represents a standalone production plant where the cellulose fraction, which was previously being used for steam/power generation, is now used for BDO production. This, however, requires an additional step of enzymatic hydrolysis to convert the cellulose fraction to glucose before it can be used for BDO production.^10^ This scenario thus allows full utilization of the carbohydrate fractions of SCB toward product formation, with only the lignin fraction being sent to the boiler.

### Process Economics

The hypothetical plant is situated in Maharashtra, India. Hence, the corresponding material costs and hourly wage rates are applied. However, the currency used for the economic assessment is US$ for easy comparison with the literature. The costs are adjusted to the year 2021 based on Chemical Engineering Plant Cost Index. The main economic inputs and assumptions adopted for economic analysis are presented in Table 1.

**Table 1:** Economic Parameters and Assumptions

parameter	value
year of analysis	2021
project economic life	20 years
discount rate	7%
depreciation calculation method	straight line
income tax	30%

Total capital investment (TCI) for the plant is calculated by adding direct fixed capital (DFC) cost, working capital cost, and startup cost. DFC is based on direct costs, indirect costs, and contractor’s fee and project contingency, which are taken as 5 and 10% of the sum of direct and indirect costs. Direct cost is made up of equipment purchase cost and costs related to piping, instrumentation, insulation, electrical facilities, buildings, and yard improvement which are 40, 15, 6, 12, 15, and 10% of total equipment purchase cost, respectively. The indirect cost includes engineering and construction costs, which are both 10% of direct costs. Working capital is estimated based on expenses to cover 15 days of raw materials and 30 days of labor, utilities, and waste treatment. The startup cost is calculated as 5% of DFC.

The total operating cost of the plant is calculated by adding costs associated with raw materials, labor, waste treatment/disposal, utilities, facility maintenance, depreciation, and miscellaneous costs, including insurance and local taxes. Additionally, we need to include the overhead cost incurred by the operation of non-process-oriented facilities and organizations, such as accounting, payroll, fire protection, security, cafeteria, etc., which are all clubbed together as factory expenses. The unit cost of individual raw materials, chemicals, and utilities used in the process is described in Table 2.

**Table 2:** Raw Material, Chemical, and Utility Cost

parameter	cost	units	source
feedstock	50	US$/MT	(17)(23)(35)
ammonium sulfate, (NH_4_)_2_SO_4_	0.1	US$/kg	(35)
DAP, (NH_4_)_2_HPO_4_	0.1	US$/kg	(36)
EDTA disodium	1.65	US$/kg	(37)
sulfuric acid	0.07	US$/kg	(36)
electricity price	0.077	US$/kW h	(26)
cooling water	0.032	US$/1000 L	(26)
lime	0.07	US$/kg	(21)
process water	0.27	US$/kg	(18)
oleyl alcohol	0.982	US$/kg	(38)
natural gas	1.86	US$/MMBtu	(23)
inoculum	0.006	US$/kg	(26)
steam	0.018	US$/kg	(26)

### Profitability Analysis

The net present value (NPV), a measure of establishing a project’s potential profitability, is calculated assuming a discount rate of 7% and 20 years project economic life. Positive NPV suggests that the plant will see financial gains over its lifetime and establish its economic feasibility to justify the present-day investment. NPV, payback time, and return of investment (ROI) were calculated to assess the profitability of BDO production from SCB in the proposed biorefinery. The BDO selling price was assumed to be US$ 3/kg.^11−13^

### Sensitivity Analysis

Any change in economic parameters, such as cost of feedstock, utility cost, BDO selling price, discount rate, etc., could potentially affect the final economic performance of the proposed biorefinery. A sensitivity analysis was thus conducted for a % change in these parameters, and the corresponding NPV in each scenario was calculated.

## Results and Discussion

### Total Capital Investment

Estimates of TCI for the 96 MT/day SCB processing plant under the five scenarios are presented in Table 3. These are accrued by adding DFC, working capital, and start-up costs for each unit in the plant. For simplicity, the whole plant was divided into four different processing stages, including pre-treatment, fermentation, DSP, and utilities. Here, pre-treatment refers to all processes required to obtain detoxified hydrolysate, starting from SCB size reduction, steam explosion, washing, screw pressing, and detoxification. Fermentation refers to pasteurization, seeding inoculum, and then fermentation of xylose/glucose to BDO. DSP refers to processes starting from the fermentation broth and centrifugation, followed by HED to obtain BDO (99%). The utility section refers to the boiler for steam production and steam turbine for power generation. The solid residue from the screw-press unit was used as a fuel to the boiler in scenarios 1 and 2. For scenarios 3, 4, and 5, along with the solid residue, additional SCB was also added to the boiler as fuel to meet the total steam requirement.

**Table 3:** Summary of TCI of the BDO Production Plant for the Five Scenarios

	scenario 1	scenario 2	scenario 3	scenario 4	scenario 5
section name	annexed to sugar mill	annexed to sugar mill	standalone facility	standalone facility	standalone facility
direct fixed capital cost (million US$)					
pre-treatment	2.522	2.522	2.522	2.522	0.000
fermentation	16.928	16.928	16.928	16.928	22.284
DSP	1.844	1.844	1.844	1.844	1.637
utilities	0.000	0.000	2.815	2.815	0.911
working capital (million US$)					
pre-treatment	0.008	0.080	0.080	0.152	0.000
fermentation	0.019	0.019	0.124	0.124	0.194
DSP	0.009	0.009	0.012	0.012	0.021
utilities	0.005	0.005	0.006	0.014	0.002
start-up cost (million US$)					
pre-treatment	0.126	0.126	0.126	0.126	0.000
fermentation	0.846	0.846	0.846	0.846	1.114
DSP	0.092	0.092	0.092	0.092	0.082
utilities	0.000	0.000	0.141	0.141	0.046
total capital investment (million US$)					
pre-treatment	2.656	2.728	2.728	2.800	0.000
fermentation	17.793	17.793	17.898	17.898	23.592
DSP	1.946	1.946	1.949	1.949	1.740
utilities	0.005	0.005	2.962	2.970	0.958
**total (million US$)**	**22.401**	**22.473**	**25.537**	**25.617**	**30.597**

As can be seen from Table 3, with a contribution of close to 95%, DFC accounts for the largest share of TCI in all five scenarios. For the first four scenarios, fixed capital costs for the pre-treatment, fermentation, and DSP remain the same, and costs related to utilities only appear in scenarios 3 and 4. For scenario 5, costs in all stages of the process are different compared to other scenarios, mainly due to additional equipment costs associated with enzymatic hydrolysis (pre-treatment). DFC in the fermentation stage for scenario 5 is also higher compared to other scenarios as the number of staggered units increases for fermentation of the additional glucose that is generated in pre-treatment. Overall, DFC for scenarios 1 and 2 is the same (US$ 21,294,035), while that of scenarios 3 and 4 is 13% higher, and for scenario 5 is 35% higher.

Among the four stages, the biggest contributor to DFC is fermentation, covering about 80% of costs in scenarios 1 and 2, close to 70% in scenarios 3 and 4, and about 78% in scenario 5. The high DFC costs in fermentation are mainly originating from the high equipment costs of fermenters. Looking closely at working capital, we can see that these costs are lowest in scenario 1 (US$ 42,085) and increase successively as we move from scenario 2 to 5. Start-up costs, which are directly dependent on DFC, are again lowest for scenarios 1 and 2 (US$ 1,064,702), and the percentage increase for scenarios 3, 4, and 5 is similar to that observed for DFC.

TCI for the BDO production plant under scenario 1 is US$ 22,400,821. Since the contribution of working capital to the total TCI is quite less, the large variations in working capital are not transferred to TCI. Therefore, we observe less than 1% increase in TCI for scenario 2 when compared to scenario 1. However, with the additional costs associated with utilities, scenarios 3 and 4 show a jump of about 14% in TCI compared to scenario 1. For scenario 5, costs associated with additional processing result in a significant increase in TCI (US$ 30,597,140), which is 37% higher than that in scenario 1.

**Figure 2:**
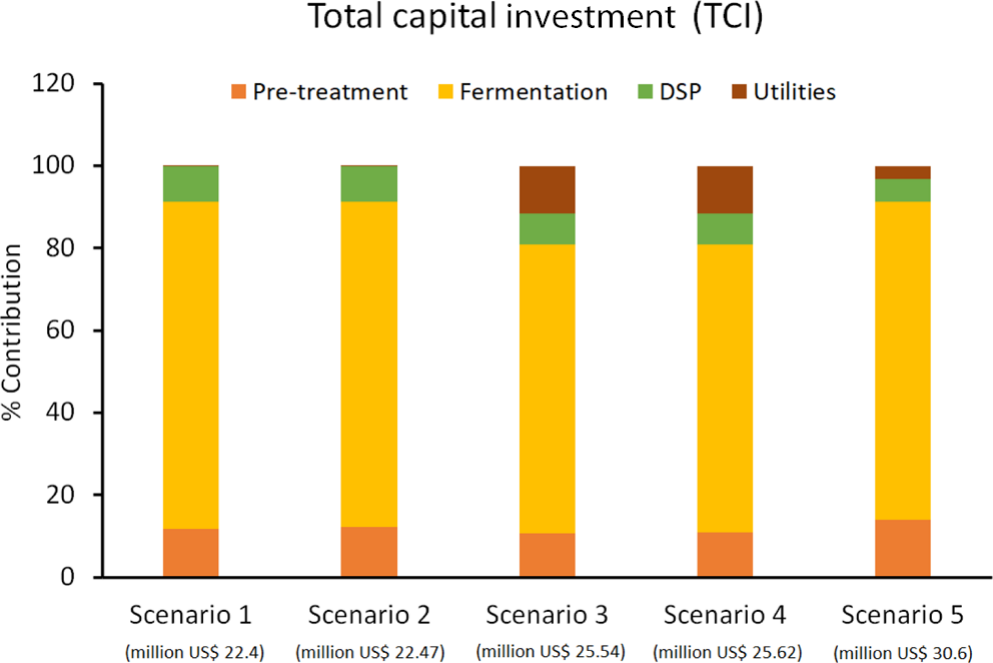
Percentage contributions of different processing stages toward TCI. The number in bracket below each scenario represents the value of TCI.

The individual percentage contributions of the four processing stages to TCI in the five scenarios are shown in Figure 2. As can be seen, for scenarios 1 and 2, the fermentation stage is the biggest contributor to TCI, about 80% in both cases, with pre-treatment and DSP costs contributing about 12 and 8%, respectively. For scenarios 3 and 4, utilities become the second biggest contributors to TCI (after fermentation), with contributions of about 12% in each case. These added equipment costs for utilities contribute to the overall 14% increase in TCI for scenarios 3 and 4. For scenario 5, pre-treatment with 14% is the second largest contributor to TCI (fermentation being the first again). This is expected due to the increased cost of pre-treatment equipment for converting cellulose to glucose in scenario 5.

### Operating Cost

**Figure 3:**
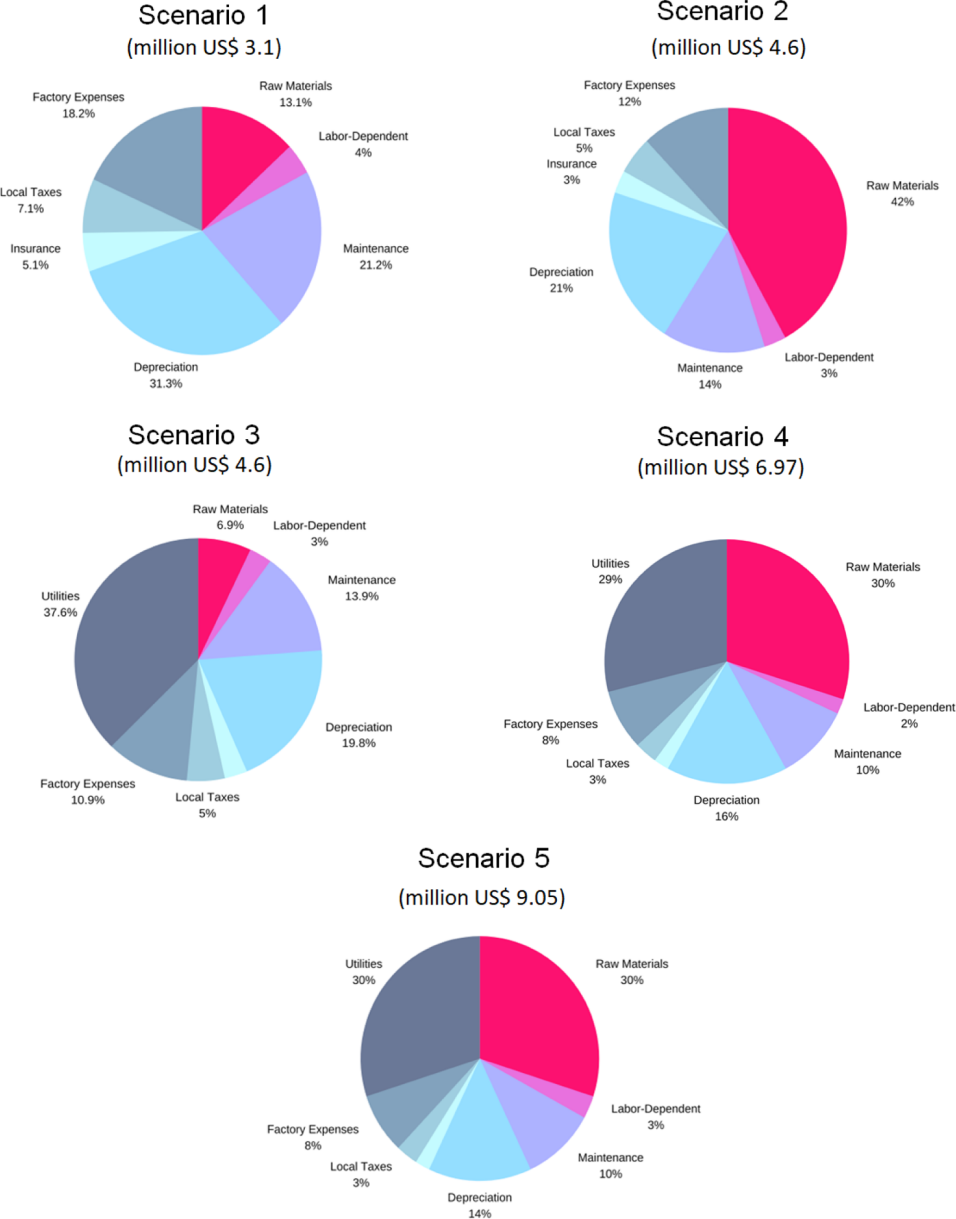
Percentage contribution of different components toward AOC for the five scenarios.

For the five scenarios, the breakdown of annual operating cost (AOC) for the fermentative production of BDO is illustrated in Table 4. As can be seen here, AOC for scenario 1 is US$ 3,056,000 and increases by about 50, 75, 130, and 200% in scenarios 2, 3, 4, and 5, respectively. Percentage contributions of individual cost components to AOC are shown in Figure 3, and this result reveals that the biggest contributor varies a lot depending on the mode of plant operation.

**Table 4:** Summary of Annual Operating Costs in Million US$ for the BDO Production Plant under Five Scenarios

cost item	scenario 1	scenario 2	scenario 3	scenario 4	scenario 5
raw materials	0.387	1.971	0.387	2.059	2.746
labor-dependent	0.133	0.133	0.133	0.133	0.271
maintenance	0.639	0.639	0.723	0.723	0.862
depreciation	0.958	0.958	1.085	1.085	1.251
insurance	0.149	0.149	0.169	0.169	0.201
local taxes	0.213	0.213	0.241	0.241	0.287
factory expenses	0.563	0.563	0.563	0.563	0.718
waste treatment/disposal	0.009	0.009	0.009	0.009	0.010
utilities	0.005	0.005	1.986	1.986	2.704
**total (million US$)**	**3.056**	**4.640**	**5.296**	**6.968**	**9.050**

For scenario 1, depreciation, maintenance, and factory expenses are the major cost contributors, with 31, 21, and 19% contributions to the total AOC. Raw materials only account for about 13% of AOC in scenario 1, mainly because the feedstock SCB is assumed to be obtained free in this case. As opposed to this, raw materials become the major contributor (42%) to AOC in scenario 2, largely due to the procurement cost of SCB. This drives the total AOC of scenario 2 to increase 1.5 times compared to scenario 1 to a value of US$ 4,640,000. Depreciation, maintenance, and factory expenses become the second, third, and fourth highest contributors.

When considering a standalone facility that is no longer annexed to the sugar mill, as described in scenario 3, the cost of utilities as expected becomes the major contributor to AOC, accounting for almost 38% of the total, again followed by depreciation, maintenance, and factory expenses. This decentralization, where utilities can no longer be accessed from the sugar mill, leads to about 1.7 times increase in AOC compared to the annexed facility in scenario 1.

For scenario 4, which considers procurement of SCB in addition to being a decentralized standalone facility, the high costs of raw materials (30%) and utilities (29%) dwarf all the other contributions. This factor also leads to an overall increase of AOC by about 2.3 times compared to scenario 1.

With US$ 9,050,000, the AOC of scenario 5 is the highest compared to all previous scenarios. Similar to scenario 4, the cost of raw materials (30%) and utilities (30%) are the major contributors, followed by depreciation (14%). The cost of enzymes for converting cellulose to glucose leads to an increase in the cost of raw materials, and overall increase in system volume drives up the expenses on utilities.

### Profitability Analysis

**Figure 4:**
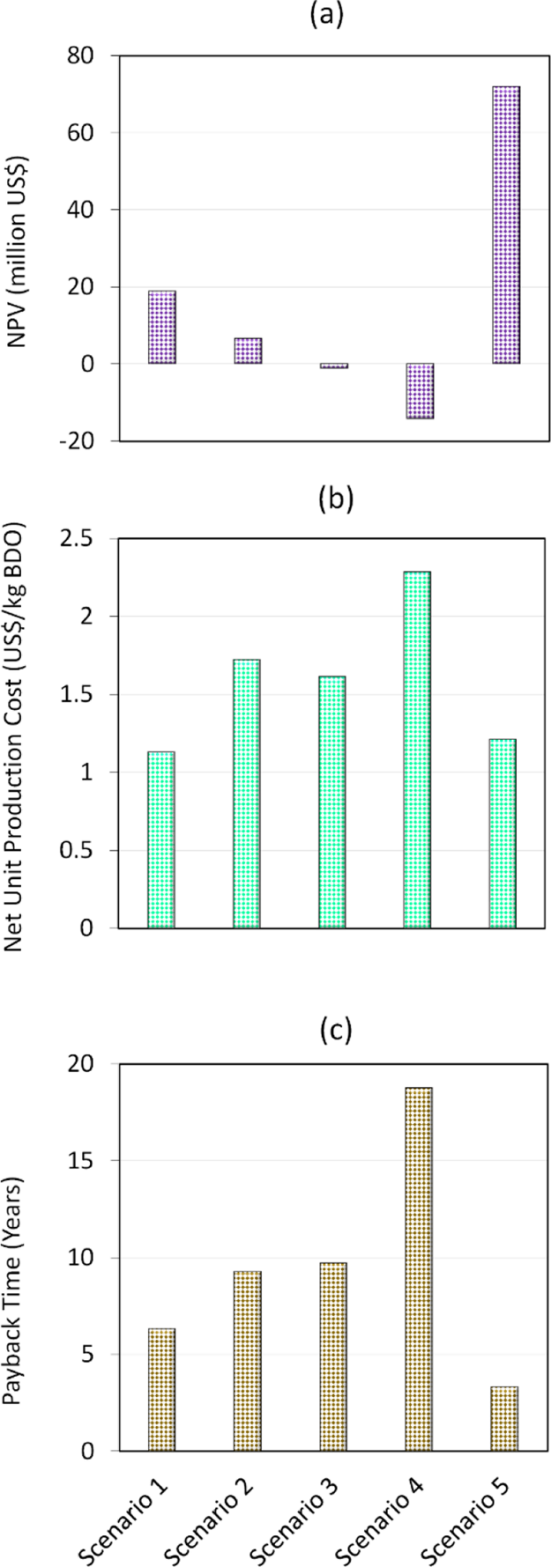
Results of profitability analysis, showing the variation of (a) NPV, (b) net unit production cost, and (c) payback time, for the five scenarios.

The main revenue of the plant comes from the sale of BDO, and in scenarios 3, 4, and 5, additional savings are accrued with the generation of power which is recycled back to the plant. The detailed economic performance of the different scenarios is illustrated in detail in Table 5. Also, the variation in NPV, unit production cost, and payback time for the five scenarios has been described in Figure 4. It should be noted that the MSP values reported in Table 5 have been calculated based on economic parameters used by Koutinas et al. (2016) for easy comparison.^13^

**Table 5:** Economic Analysis Results for the Five Scenarios

	scenario 1	scenario 2	scenario 3	scenario 4	scenario 5
total capital investment (million US$)	22.401	22.473	25.537	25.617	30.597
annual operating cost (AOC) (million US$)	3.056	4.640	5.296	6.968	9.050
BDO sale (main revenue) (million US$)	8.097	8.097	8.097	8.097	21.923
standard power generation (savings) (million US$)	0	0	0.822	0.822	0.229
net unit production cost (US$/kg BDO)	1.130	1.720	1.610	2.280	1.210
minimum selling price, MSP (US$/kg BDO)	2.370	2.960	3.370	3.990	1.860
net profit (after taxes) (million US$)	3.529	2.420	2.630	1.367	9.171
return on investment (%)	15.760	10.770	10.300	5.330	29.990
payback time (years)	6.400	9.280	9.700	18.700	3.330
**NPV (@ 7.0% discount rate) (million US$)**	**18.840**	**6.589**	**–1.155**	**–14.156**	**71.914**

As can be seen from Table 5, for scenario 1, where the BDO production plant is annexed to a sugar mill and SCB is assumed to be provided free, the net annual profit is US$ 3,529,000, with payback time and NPV of 6.4 years and US$ 18,840,000, respectively. For scenario 2, when the cost of feedstock is accounted, the increase in overall operating costs leads to 30% decrease in annual profits, resulting in 65% decrease in NPV and increases payback time to 9.3 years. Comparing scenarios 1 and 2, both of which have BDO production plant annexed to a sugar mill, it is clear that scenario 1 is more profitable, with a ROI close to 16%. With a positive NPV of US$ 6,589,000, scenario 2 will also see financial gains in the future, but its ROI is lower (∼11%).

Compared to production in an annexed facility (as in scenarios 1 and 2), moving the plant to a standalone decentralized location results in substantial drop in NPV to negative values for scenarios 3 and 4. It is largely due to the increased capital investment and operating costs. Looking at scenario 3 specifically, the net unit production cost of BDO is lower than that for scenario 2 due to the power savings. However, these savings are still not sufficient to offset the increased investment and operating costs which result in overall negative NPV (−US$ 1,155,000). Results for scenario 4 are even less favorable as the higher costs involved in SCB procurement and utilities lead to a highly negative NPV (−US$ 14,156,000), deeming the plant economically unviable.

Scenario 5, on the other hand, shows favorable economic performance (30% ROI, 3.33 years payback time, and US$ 71,914,000 NPV), proving that it is possible to turn a standalone facility profitable by utilizing all carbohydrate fractions of SCB for BDO production. With a more useable substrate, the higher overall yield of BDO leads to increased revenue (2.7 times) compared to the first four scenarios, which helps in outweighing the higher capital investment and operating costs associated with scenario 5, making it economically feasible even if SCB and utilities are not available for free.

Previously, Koutinas et al. (2016) presented the techno-economic evaluation of BDO production via fermentation of three different carbon sources: glycerol, sucrose, and sugarcane molasses. They calculated the MSP of BDO (unit price for zero NPV) for each feedstock and reported that MSP varied from 2.1 to 2.9 US$/kg for glycerol, 1.97 to 5.26 US$/kg for sucrose, and 2.6 to 4.8 US$/kg for sugarcane molasses. When using the same economic parameters (10% discount rate, plant lifetime—30 years, and depreciation—7 years) as used by Koutinas et al. (2016), the MSP of BDO from the current plant varies from US$ 1.86/kg (for scenario 5) to US$ 3.99/kg (for scenario 4). MSPs for other scenarios of the current study are US$ 2.37/kg for scenario 1, US$ 2.96/kg for scenario 2, and US$ 3.37/kg for scenario 3. Thus, scenario 5 in this proposed BDO production plant using SCB shows better economic viability than BDO production using either glycerol, sucrose, or sugarcane molasses.^13^

Some studies have reported TEA results based on unit production cost. For example, Mailaram et al. (2021) described a techno-economic assessment for BDO production from BSG with a plant capacity of 100 MT/day. They reported unit production costs to be in the range of US$ 1.736/kg to US$ 1.842/kg. Current analysis based on fermentative BDO production using SCB with a plant capacity of 96 MT/day [which is comparable to the one used by Mailaram et al. (2021)] predicts the unit production cost to be in the range of US$ 1.13 US$/kg (scenario 1) to US$ 2.28/kg (scenario 4). Mailaram et al. (2021) also showed that economies of scale would reduce the production cost to US$ 1.07/kg if the plant capacity was increased 20 times (2000 MT/day).^26^ A similar decrease in unit production cost could be achieved for the plant proposed in this work. For example, if the plant capacity for the SCB to BDO plant is hypothetically increased to 2000 MT/day, the unit production cost of BDO in scenario 1, which was 1.13 US$/kg (for 96 MT/day capacity), would fall to US$ 0.48/kg. These numbers are very promising; however, it should be noted that the decision for the real plant capacity would depend on several factors such as local availability of the feedstock, regulations, space available for expansion, etc. Therefore, these decisions would require further deliberation.

Benalcázar et al. also performed economic assessments for the production of bio-based BDO from the syngas platform involving gasification of lignocellulosic biomass (such as pine, corn stover, SCB, and eucalyptus) followed by syngas fermentation. They reported the MSP of BDO varied between US$/kg 2.75 and US$/kg 2.9 using this hybrid process (biomass gasification followed by syngas fermentation). The results from our study show that with complete utilization of carbohydrate fractions, BDO produced using microbial fermentation of SCB could be more profitable than using the hybrid syngas platform,^39^ Maina et al. (2019) worked on optimization of BDO production in fed-batch cultures using very high polarity sugar from sugarcane mills and also presented techno-economic evaluations. Based on the analysis, they estimated BDO MSP to be varying between US$ 3.12/kg and 2.67/kg for annual production capacities of 10,000 and 50,000 MT, respectively.^40^ Compared to Maina et al. (2019), microbial BDO production from SCB in the current work with annual production capacity of 29,952 MT (96 MT/day, 312 operating days) predicts a much lower MSP of BDO US$ 1.86/kg (scenario 5).

Zang et al. (2020) also conducted a detailed TEA for conversion of switchgrass to BDO and co-products such as furfural and technical lignin, enabled by high-solid loading deep eutectic solvent (DES) pre-treatment. Based on their analysis, MSP of BDO was estimated to be US$ 1.7/kg–1.74/kg, depending on the solid loadings during the process.^41^ This reported MSP is only slightly below the lowest MSP (US$ 1.86/kg for scenario 5) obtained in our analysis. Also, it should be noted that economic analysis of Zang et al. (2020) is based on plant life assumption of 30 years, whereas the same in our proposed plant is assumed to be 20 years.

The effectiveness and economic viability of SCB as a potential feedstock for fermentative BDO production are thus highlighted when compared to both bio-based approaches as well as conventional fossil-derived BDO from previous studies.

### Sensitivity Analysis

The above analysis is largely dependent on the specific economic parameters selected for the study. Therefore, sensitivity analysis is performed to understand the effect of variation in important parameters, such as cost of SCB, selling price of BDO, utility cost, plant operational life, and discount rate, on the economic performance of the different scenarios.

**Figure 5:**
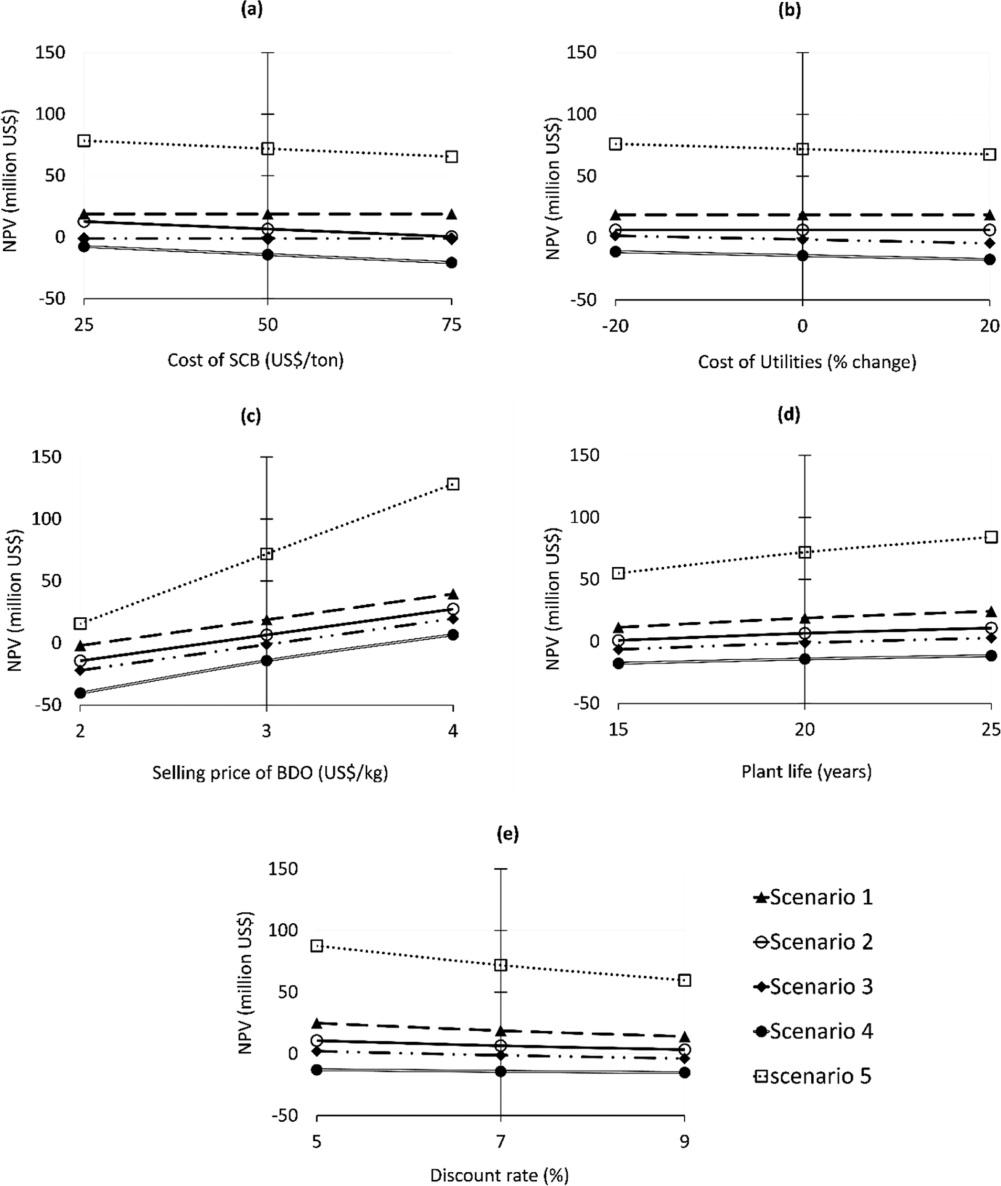
Sensitivity of plant NPV to changes in technical and economic parameters, (a) cost of SCB (US$/ton), (b) cost of utilities (% change), (c) selling price of BDO (US$/kg), (d) plant life (years), and (e) discount rate (%).

**Figure 6:**
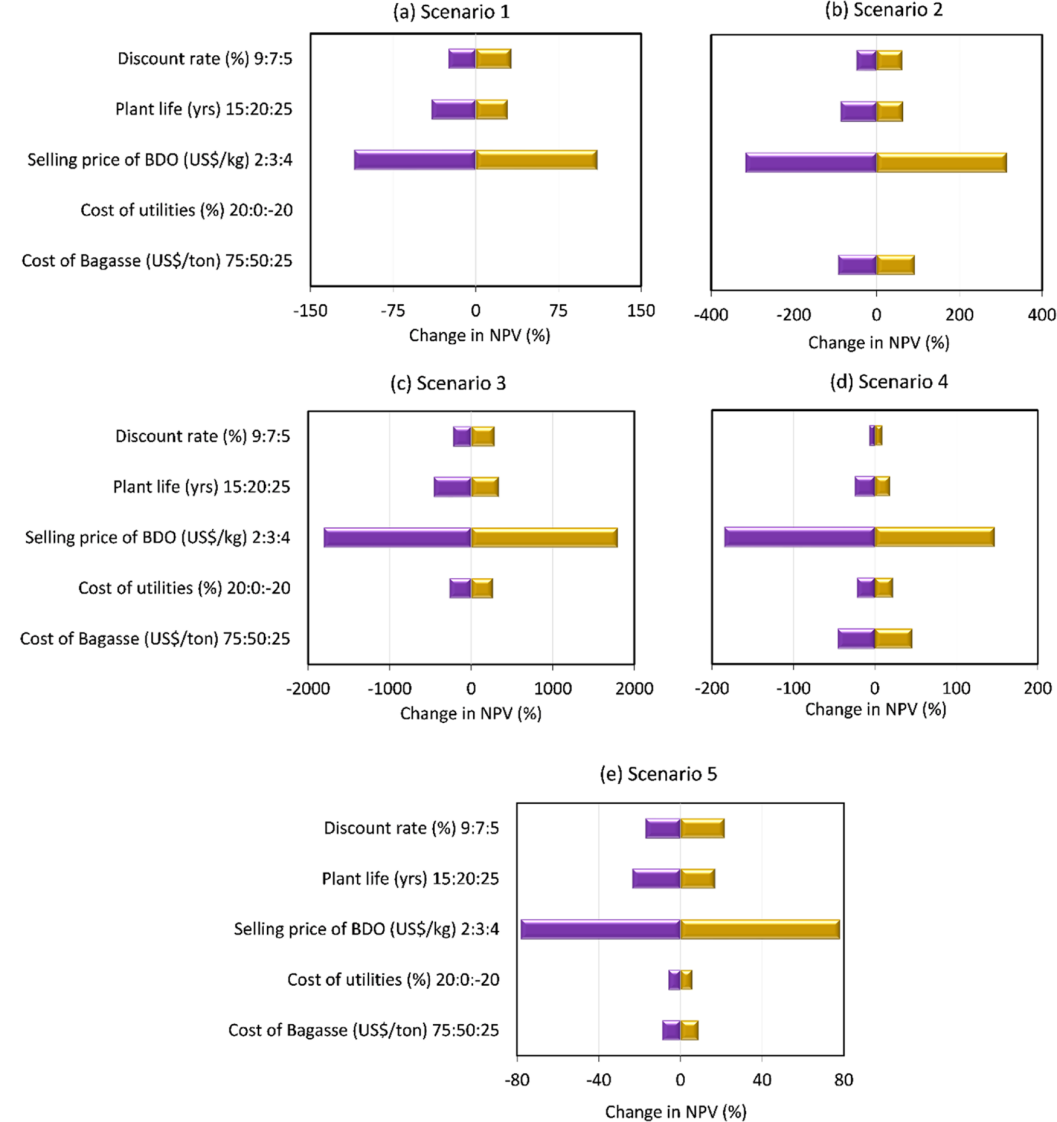
Percentage change in NPV with respect to the base condition for different technical and economic parameters varied for (a) scenario 1, (b) scenario 2, (c) scenario 3, (d) scenario 4, and (e) scenario 5.

The results of this sensitivity analysis are presented in Figure 5, which shows absolute change in NPV for the five scenarios as a function of change in parameters. Figure 6 shows percentage change in NPV with respect to the original values for the five scenarios and allows us to understand the largest influencing factors.

For the first case, as can be seen in Figure 5a, economic performance is evaluated considering three different purchase costs for SCB, US$ 25, 50, and 75/MT. This is important because the price of SCB is not fixed and depends on the country, produce, local regulations, etc.^16,42^ It can be seen that changes in the SCB cost only affect the NPV of scenarios 2, 4, and 5 because SCB was considered to be free in scenarios 1 and 3. As expected, NPV was found to decrease with increasing SCB cost. For scenario 2, as the cost of SCB increases to US$ 75/MT, the estimated NPV of the plant goes down, reaching a low value of US$ 463,470. It is clear that even a small further increase in SCB price would lead to a negative NPV and make the project economically unsustainable. For scenario 4, the decrease in SCB cost to US$ 25/MT improves the performance, but it is still not sufficient to make the plant economically viable. Scenario 5 maintains large positive NPV (US$ 65,449,131) even when the SCB cost is increased to US$ 75/MT.

It can be seen from Figure 6 that cost of SCB has the most influence on scenario 2 which shows about ±90% change in NPV, followed by scenario 4 which shows around ±45% change, with the least influence shown in scenario 5, for which the NPV only shows about ±9% change when cost of SCB is varied between 25 US$/MT and 75US$/MT.

In the second case (Figure 5b), the costs of all the utilities used in the process were varied ±20% from their original values. In the first two scenarios, the utilities are assumed to be provided by the sugar mill. Hence, the change in utility cost only influences the NPV of scenarios 3, 4, and 5. For scenario 3, NPV, which was negative in the original case, reaches a positive value (US$ 1,929,699) when the utility costs are reduced by 20%. This would suggest that even a standalone facility of fermentative BDO production from xylose derived from SCB can be economically feasible if the costs of utilities can be subsidized by 20%. Scenario 4, on the other hand, remains economically unfavorable even after reducing utility costs by 20%. For scenario 5, increasing the cost of utilities by 20% has very little effect on the NPV, which remains much higher than all other scenarios. Looking at percentage change in NPVs with respect to the cost of utilities, it can be seen in Figure 6 that scenario 3 is most affected, showing ±270% change, whereas this parameter has little influence on scenarios 4 and 5 which only show ±22 and ±5% change in NPVs, respectively.

The third factor, i.e., the selling price of BDO, has a very strong linear impact on the NPV of the plant. It can be seen in Figure 5c that all scenarios, except scenario 5, show a negative NPV when the BDO selling price is reduced to US$ 2/kg. On the other hand, standalone facilities described in scenarios 3 and 4, which showed negative NPV at the original BDO selling price of US$ 3/kg, become economically favorable (with a positive NPV) when the selling price is increased to US$ 4/kg. For scenario 5, NPV falls by almost 80% when the BDO selling price is reduced to US$ 2/kg, but still registers a positive NPV (US$ 15,632,793), suggesting the plant will remain economically feasible. This is important because it is possible that market forces could drive down the selling price of BDO. As can be seen in Figure 6, BDO selling price is the one parameter which has the most influence on NPV of all five scenarios, particularly for scenario 3, which shows 1800% decrease in NPV when the selling price of BDO is reduced to US$ 2/kg. Such a large percentage change is mainly due to the low starting value of NPV in the base case (with BDO selling price as US$ 3/kg) −million US$ 1.155. On decreasing the selling price of BDO to US$ 2/kg in scenario 3, it leads to a significantly negative NPV of −million US$ 22.03, whereas increasing the selling price of BDO to US$ 4/kg takes the NPV to million US$ 19.6. For all other scenarios as well, a small deviation in the BDO selling price shows a major change in NPV. Therefore, it will be critical for all plant investment decisions to get an accurate estimate of the market potential and higher limit of selling price for the product, BDO (and account for its variation in the market), before delving into detailed profitability calculations.

For a fermentation product to classify as a platform chemical, the MSP should reach 1 US$/kg.^13,43^ For scenario 5, when the BDO selling price is reduced to US$ 1/kg, NPV falls to a negative 46.8 million US$, implying that the plant would no longer be economically viable. Therefore, for the current plant capacity with the reported yield and productivity, fermentative BDO from SCB would not qualify as a platform chemical even if both cellulose and hemicellulose fractions are utilized.

The fourth factor, i.e., plant operational life, was changed ±5 years from the original assumption of 20 years (Figure 5d). It can be seen that plant life also has a strong linear influence on all scenarios. When plant life is reduced to 15 years, NPVs of scenarios 1 and 2 drop by 40 and 88%, respectively, but they remain positive, nevertheless. Scenario 3, which showed a negative NPV in the base case of 20 years, shows a positive NPV of US$ 2,754,171 when the plant life is increased to 25 years. On the other hand, ±5 years change in operational life does not result in any significant change in performances of scenarios 4 and 5 (% change of less than ±25%), and these scenarios remain economically unviable and viable, respectively, as in the original case.

The fifth factor, i.e., discount rate, is changed ±2 from the originally assumed value of 7%. As can be seen in Figure 5e, it is also shown to have an influence on all scenarios, with lower discount rates leading to higher NPVs. While the economic feasibility of scenarios 1, 2, 4, and 5 is not affected much by this change, scenario 3 (which was economically unviable in the original case) registers a positive NPV (US$ 2,176,929) when the discount rate is reduced to 5%. This represents an increase in NPV of almost 300%. Increasing the discount rate to 9% also affects scenario 3, which shows around 220% reduction in its NPV.

## Conclusions

BDO is a versatile chemical with huge derivative potentials and has promising fuel properties for blending with motor fuels. Furthermore, high BDO titers make fermentative production feasible from biomass-derived sugars. This work demonstrates the techno-economic feasibility of BDO production from SCB. The process model is developed for processing 96 MT of SCB per day under five different scenarios. Overall looking at the profitability and sensitivity analysis, a standalone facility for BDO production from SCB could show financial gains and become economically feasible if both cellulose and hemicellulose fractions are utilized for BDO production. If BDO is to be produced only using xylose derived from SCB, it may still be possible to run an economically viable plant if either one of the conditions is met: the plant is annexed to a sugar mill which can provide the utilities, or the feedstock is free, or the cost of utilities can be subsidized, or BDO can be sold at a higher price, or plant operational life can be increased to 25 years, or if the discount rate can be lowered to 5%.

Among the five plant scenarios considered in this work, scenario 1 predicted the lowest unit production cost for BDO, 1.13 US$/kg, while scenario 5 showed the best performance in terms of economic viability with ROI of about 30% and NPV of almost 72 million US$. It was also seen that compared to other scenarios, scenario 5 was least susceptible to any changes in the selected economic parameters. The future work will be directed toward the techno-economic feasibility studies and life cycle analysis for expanding this plant to convert BDO derived via a fermentative route to higher value chemicals such as 1,3-butadiene and methyl ethyl ketone.

## Supporting Information Available

Detailed process data for all scenarios (process conditions and mass flow rates), simplified process schemes, resource demand breakdown, equipment costs, and BDO fermentation conditions (PDF)
