# Supplementary material for: Techno-Economic Analysis of 2,3-Butanediol Production from Sugarcane Bagasse
Source: ACS Sustain Chem Eng. 2023 May 22;11(22):8337–49. doi: 10.1021/acssuschemeng.3c01221 (PMC10245391; doi:10.1021/acssuschemeng.3c01221)
Supplement: Supplementary file 1 — sc3c01221_si_001.pdf [file sc3c01221_si_001.pdf]

Supporting Information

Techno-economic analysis of 2,3-butanediol (BDO) production from sugarcane bagasse

Siddharth Gadkari[a,*], Vivek Narisetty[b], Sunil K. Maity[c], Haresh Manyar[d], Kaustubha Mohanty[e], Rajesh Banu Jeyakumar[f], Kamal Kishore Pant[g], Vinod Kumar[b,*]

[a] *Department of Chemical and Process Engineering, University of Surrey, Guildford GU2 7XH, United Kingdom*
[b] *School of Water, Energy and Environment, Cranfield University, Cranfield MK43 0AL, United Kingdom*
[c] *Department of Chemical Engineering, Indian Institute of Technology Hyderabad, Sangareddy 502284 Telangana, India*
[d] *School of Chemistry and Chemical Engineering, Queen's University Belfast, Belfast BT9 5AG, Northern Ireland, United Kingdom*
[e] *Department of Chemical Engineering, Indian Institute of Technology Guwahati, Guwahati, Assam 781039, India*
[f] *Department of Life Sciences, Central University of Tamil Nadu, Neelakudi, Thiruvarur, Tamil Nadu 610005, India*
[g] *Department of Chemical Engineering, Indian Institute of Technology Delhi, New Delhi 110016, India*

***Corresponding authors**

Siddharth Gadkari; Email: s.gadkari@surrey.ac.uk

Vinod Kumar; E-mail: Vinod.Kumar@cranfield.ac.uk

Number of pages: 8

Number of Tables: 6

Number of Figures: 2

Table S1. Process conditions and mass flow rates for representative streams of the BDO production plant in scenarios 1-4 (see Figure S1).

STREAM	1	2	3	4	5	6	7	8	9	10
**Stream Properties**										
Temperature (°C)	25	25	25	187.09	100	101.17	66.26	66.26	66.26	75.87
Pressure (bar)	1.01	1.01	1.01	12	1.01	1.01	1.01	1.01	1.01	1.01
**Component Flowrates (MT/h)**										
Ash	0.136	0.136	0	0	0.136	0.136	0.109	0.109	0	0.027
Cellulose	1.94	1.94	0	0	1.746	1.746	1.746	1.746	0	0
Hemicellulose	0.84	0.84	0	0	0	0	0	0	0	0
Lignin	0.72	0.72	0	0	0.72	0.72	0.612	0.612	0	0.108
Other Solids	0.364	0.364	0	0	0.364	0.364	0.364	0.364	0	0
Water	2.154	2.154	0	2.003	4.105	3.582	4.729	3.027	1.702	2.759
H2SO4	0	0	0.11	0	0.11	0.11	0	0	0	0.11
Acetic-Acid	0	0	0	0	0.005	0.005	0	0	0	0.005
Furfural	0	0	0	0	0.006	0.006	0	0	0	0.006
other-sugars	0	0	0	0	0.231	0.231	0	0	0	0.231
Xylose	0	0	0	0	0.903	0.903	0.452	0.09	0.361	0.452
**TOTAL (MT/h)**	**6.154**	**6.154**	**0.11**	**2.003**	**8.326**	**7.803**	**8.012**	**5.948**	**2.064**	**3.697**

STREAM	11	12	13	14	15
**Stream Properties**					
Temperature (°C)	71.81	37	40.4	100	25
Pressure (bar)	1.01	1.01	1.01	1.01	1.01
**Component Flowrates (MT/h)**					
2,3-Butanediol	0	0.371	0.358	0.341	0.338
Ash	0.027	0.027	0.026	0.008	0
Biomass	0	0.035	0.017	0.001	0
Calcium acetate	0.006	0.006	0.006	0.002	0
CaSO4	0.152	0.152	0.147	0.044	0
Furfural	0.006	0.004	0.004	0.001	0
Lignin	0.108	0.108	0.104	0.031	0
lime	0.014	0.014	0.014	0.004	0
other-sugars	0.231	0.069	0.067	0.02	0
Water	4.5	4.693	4.53	1.359	0
Xylose	0.813	0.174	0.168	0.05	0
Oleyl alcohol	0	0	0	0.24	0.002
**TOTAL (MT/h)**	**5.857**	**5.653**	**5.441**	**2.101**	**0.34**

Table S2. Process conditions and mass flow rates for representative streams of the BDO production plant in scenarios 5 (see Figure S2).

STREAM	1	2	3	4	5	6	7
**Stream Properties**							
Temperature (°C)	25	25	25	187.09	100	101.17	69.54
Pressure (bar)	1.01	1.01	1.01	12	1.01	1.01	1.01
**Component Flowrates (MT/h)**							
Ash	0.136	0.136	0	0	0.136	0.136	0.136
Cellulose	1.94	1.94	0	0	1.746	1.746	1.746
Hemicellulose	0.84	0.84	0	0	0	0	0
Lignin	0.72	0.72	0	0	0.72	0.72	0.72
Other Solids	0.364	0.364	0	0	0.364	0.364	0.364
Water	2.154	2.154	0	2.003	4.105	3.582	7.488
H_2SO_4	0	0	0.11	0	0.11	0.11	0.11
Acetic-Acid	0	0	0	0	0.005	0.005	0.005
Furfural	0	0	0	0	0.006	0.006	0.006
other-sugars	0	0	0	0	0.231	0.231	0.231
Xylose	0	0	0	0	0.903	0.903	0.903
**TOTAL (MT/h)**	**6.154**	**6.154**	**0.11**	**2.003**	**8.326**	**7.803**	**11.709**

STREAM	8	9	10	11	12	13	14	15
**Stream Properties**								
Temperature (°C)	69.32	69.34	69.34	69.34	37	39.61	100	25
Pressure (bar)	1.01	1.01	1.01	1.01	1.01	1.01	1.01	1.01
**Component Flowrates (MT/h)**								
2,3-Butanediol	0	0	0	0	1.024	0.976	0.927	0.918
Ash	0.136	0.136	0.088	0.048	0.048	0.045	0.014	0
Biomass	0	0	0	0	0.073	0.004	0.001	0
Calcium acetate	0.006	0.006	0.001	0.005	0.005	0.005	0.002	0
$CaSO_4$	0.152	0.152	0.02	0.132	0.132	0.126	0.038	0
Furfural	0.006	0.006	0.001	0.005	0.004	0.004	0.001	0
Lignin	0.72	0.72	0.72	0	0	0	0	0
lime	0.014	0.014	0.002	0.012	0.012	0.012	0.004	0
other-sugars	0.231	0.231	0.031	0.2	0.06	0.057	0.017	0
Water	7.53	7.67	1.016	6.653	6.989	6.665	1.999	0
Xylose	0.903	0.903	0.12	0.784	0.168	0.16	0.048	0
Oleyl alcohol	0	0	0	0	0	0	0.45	0.005
Other Solids	0.364	0.364	0.048	0.316	0.316	0.301	0.09	0
Cellulose	1.746	0.175	0.175	0	0	0	0	0
Glucose	0	1.746	0.231	1.515	0.14	0.133	0.04	0
Enzyme	0	0.035	0.005	0.03	0	0	0	0
**TOTAL (MT/h)**	**11.808**	**12.158**	**2.458**	**9.7**	**8.971**	**8.488**	**3.631**	**0.923**

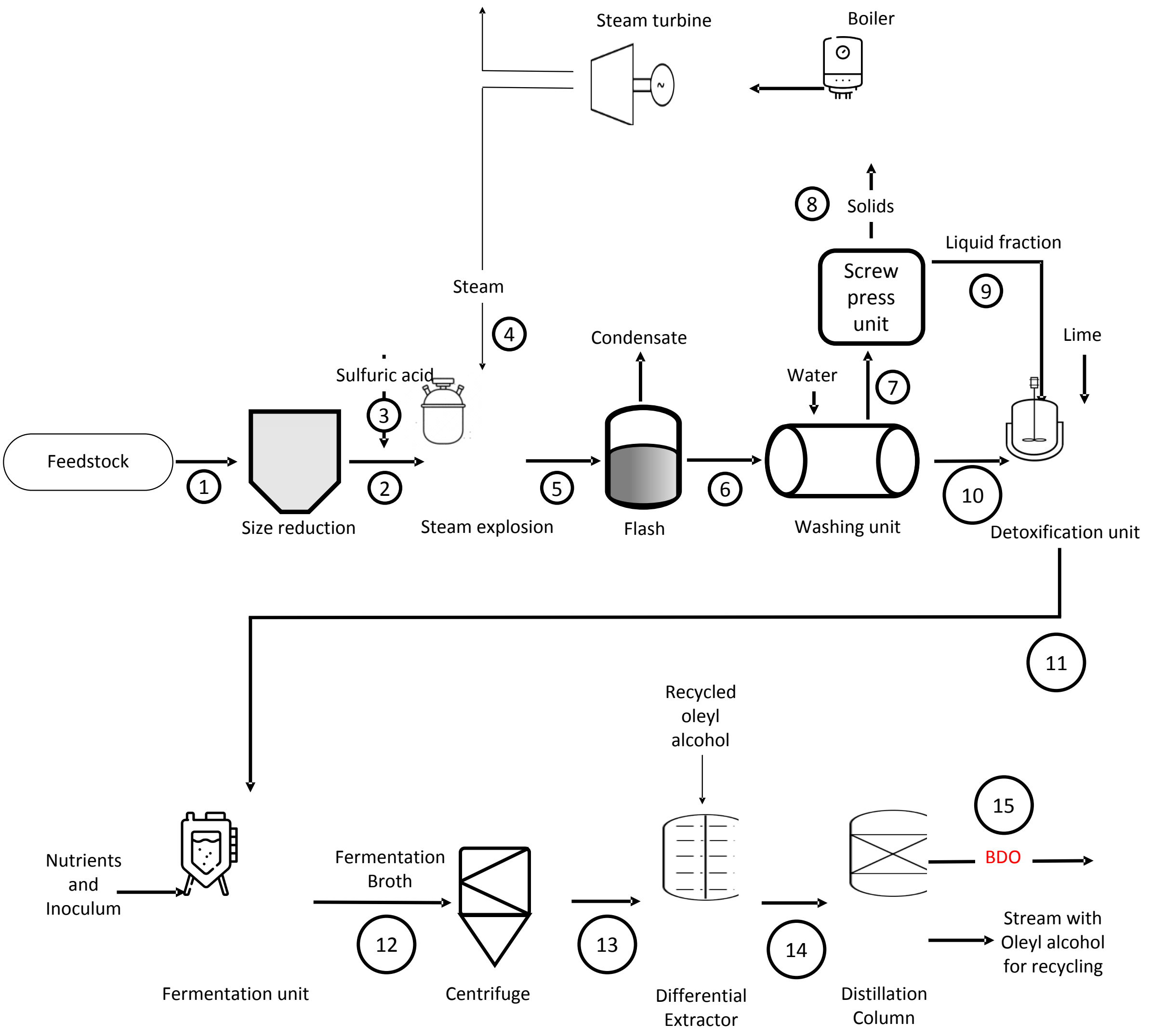

Figure S1. Simplified scheme of the BDO production plant modeled in this study for scenarios 1-4 with stream numbers for Table S1.

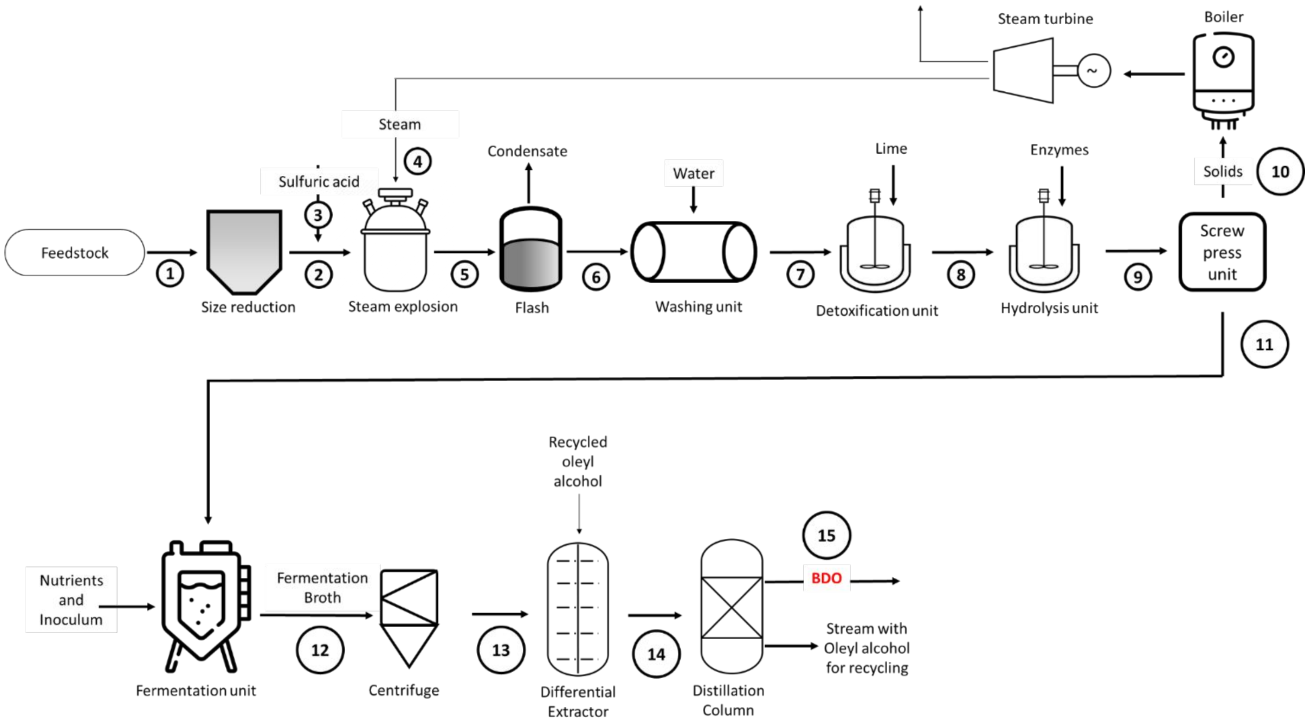

Figure S2. Simplified scheme of the BDO production plant modeled in this study for scenario 5 with stream numbers for Table S2.

Table S3. Resources demand breakdown in different processing stages of the plant for the five scenarios (+ sign signifies generation).

Resource	Section	Demand/Production	
		Scenario 1-4	Scenario 5
**Cooling Water (kg/hr)**	Pre-treatment	160236.28	160236.28
	Fermentation	255143.24	415122.27
	DSP	9116.29	25631.83
**Steam (kg/hr)**	Pre-treatment	2000	2000
	Fermentation	540.92	890
	Utilities	+ 2550	+ 2990
**Natural Gas (kg/hr)**	DSP	14.05	37.88
**Power (kW-h/hr)**	Pre-treatment	986.68	939.41
	Fermentation	1429.24	2337.11
	DSP	40.7	48.44
	Utilities	+ 1347.94	+ 375.67

Table S4. List of equipment cost (common for scenarios 1 to 4).

Description	Quantity/ Staggered	Unit Cost (US $)	Cost (US $)
Grinder<br>Rated Throughput = 6154.00 kg/h	1 / 0	100000	100000
Reactor - Steam Explosion<br>Vessel Volume = 3817.02 L	1 / 0	88000	88000
Flash Drum<br>Vessel Volume = 1152.81 L	1 / 0	8000	8000
Washer<br>Rated Throughput = 7803.17 kg/h	1 / 0	79000	79000
Screw Press<br>Throughput = 8011.50 kg/h	1 / 0	227000	227000
Steam Generator<br>Throughput = 26390.07 kg/h	1 / 0	365000	365000
Extraction Steam Turbine-Generator<br>Turbine Delivered Shaft Power = 2629.44 kW	1 / 0	642000	642000
Stirred Reactor - Detoxification<br>Vessel Volume = 6315.82 L	1 / 0	209000	209000
Pasteurizer<br>Rated Throughput = 5715.96 L/h	1 / 0	23000	23000
Seed Fermenter<br>Vessel Volume = 3.86 m3	1 / 2	40000	120000
Seed Fermenter<br>Vessel Volume = 19.93 m3	1 / 2	107000	321000
Seed Fermenter<br>Vessel Volume = 53.75 m3	1 / 2	195000	1365000
Fermenter<br>Vessel Volume = 179.29 m3	2 / 4	337000	2022000
Centrifugal Compressor<br>Compressor Power = 423.73 kW	1 / 0	537000	537000
Decanter Centrifuge<br>Throughput = 5551.17 L/h	1 / 0	273000	273000
Differential Extractor<br>Extractor Volume = 7970.36 L	1 / 0	111000	111000
Distillation Column<br>Column Volume = 96.22 L	1 / 0	40000	40000
Heat Exchanger<br>Heat Exchange Area = 64.62 m2	1 / 0	96000	96000
Heat Exchanger<br>Heat Exchange Area = 6.56 m2	1 / 0	24000	24000
Blending Tank<br>Vessel Volume = 6351.07 L	1 / 0	35000	35000
**TOTAL (US$)**			**6685000**

Table S5: BDO fermentation conditions.

Organism	Mutant strain of *Enterobacter ludwigii*
Temperature	37 °C
Pressure	1.01 bar
Number of vessels (Quantity/ Staggered)	2/4
Size (volume) of the Vessel	179.29 m^3
Solids level	163 kg/h
Residence time	24 hours

Table S6: BDO fermentation reactions and assumed conversions.

	Reaction	% Conversion
Pentose sugar to BDO	$6C_5H_{10}O_5 + 2.5O_2 \rightarrow 5C_4H_{10}O_2 + 10CO_2 + 5H_2O$	80
Other sugars to BDO	$C_6H_{12}O_6 + 0.5O_2 \rightarrow C_4H_{10}O2 + 2CO_2 + H_2O$	50
